# RECONSIDERING MANDATORY RETIREMENT FOR POLITICIANS: A MULTIDISCIPLINARY APPROACH

**DOI:** 10.1093/geroni/igae098.3248

**Published:** 2024-12-31

**Authors:** Roy Tzemah-Shahar, Aviad Menashe

**Affiliations:** University of Haifa, Haifa, HaZafon, Israel; The Open University, Israel, Ra’anana, HaMerkaz, Israel

## Abstract

The upcoming 2024 U.S. presidential election has ignited a widespread discourse on the age of candidates; globally, the political arena has witnessed seasoned leaders surpassing the age of 80 and continuing to hold office. The advanced age of politicians raises multifaceted questions across cultural, social, legal, political, and gerontological research fields. Is there a case for considering mandatory retirement age for politicians, and under what circumstances? We review available literature, federal and state laws, media reports (e.g., social networks), and health research focusing on the impact of age on work-related performance. Moreover, we explore theoretical and practical aspects of age and ageism in the workforce. The debate surrounding the advanced age of politicians has intensified in recent decades: older politicians may hinder the rise of new generations and raise concerns about their physical or cognitive ability in old age; however, these leaders are often perceived as experienced and well-connected, providing an irreplaceable wealth of knowledge. While retirement age varies globally, only a few countries have set mandatory retirement age for politicians. Recent health studies suggest that chronological age alone is insufficient to determine an individual’s ability to participate in the workforce; however, there are no objective criteria other than chronological age for mandatory retirement of most professions, including politicians. There is no consensus on ability or disability of older politicians to perform adequately, but their experience remains unparalleled. Physical and cognitive function, as reflected in biological age metrics, may serve as an alternative to chronological age concerning mandatory retirement.

